# Supplementary material for: The Use of Bi-Nasal Prongs for Delivery of Non-Invasive Ventilation to Foals
Source: Animals (Basel). 2024 Mar 11;14(6):865. doi: 10.3390/ani14060865 (PMC10967355; doi:10.3390/ani14060865)
Supplement: Supplementary file 1 [file animals-14-00865-s001.zip › animals-2888543-supplementary.pdf]

The use of nasal prongs for delivery of non-invasive ventilation to foals

Supplementary information

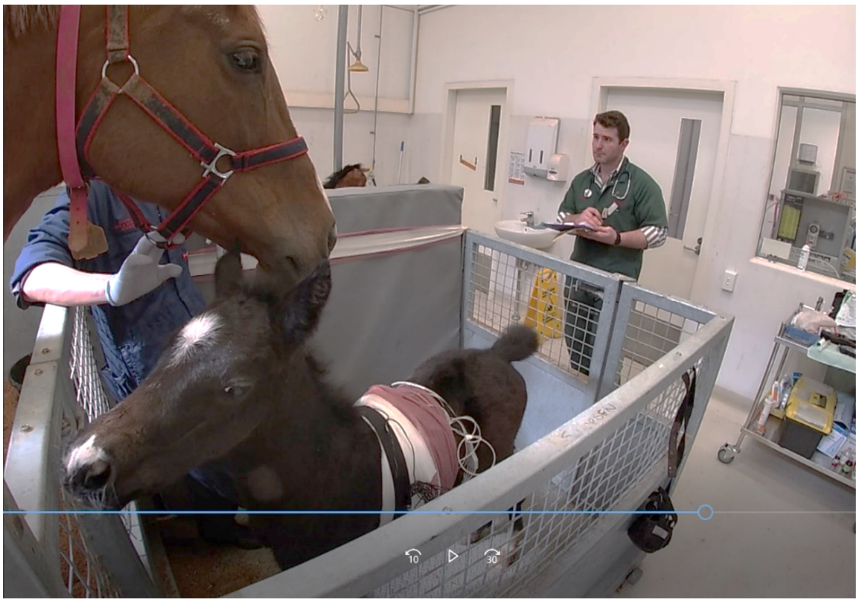

Supplementary Figure S1. Still image from video recording of F2 during T1. The foal is in the dedicated space adjacent to the mare, who is seen at the left of screen in close proximity to the foal. The foal is demonstrating tail flagging behaviour, which is recorded in real-time and by independed evlaution of the video recording, using a behavioural ethogram.

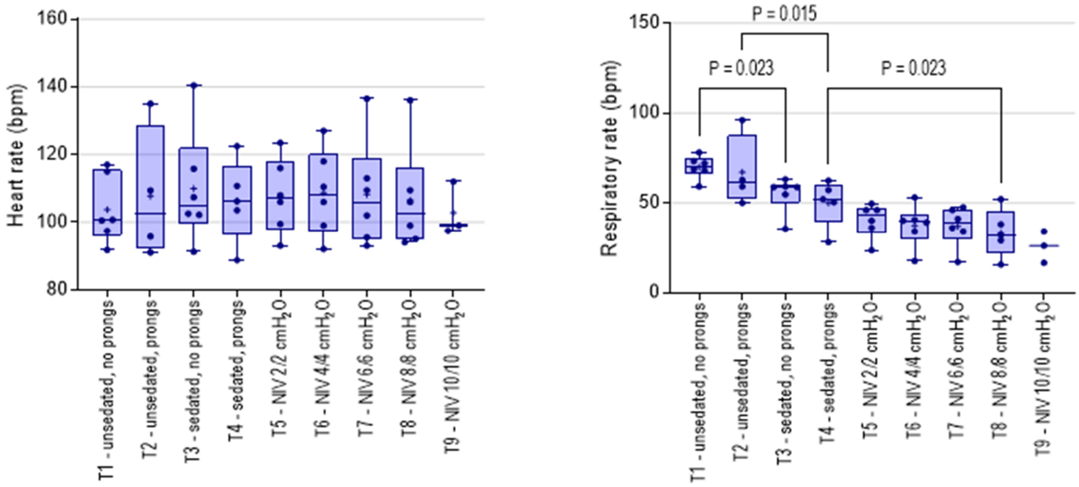

Supplementary Figure S2. Heart rate (HR) and observed respiratory rate (RRobs) during each intervention. Sedation score (left), predictably, increased after sedation and was greatest in sedated foals with nasal prongs; significant differences are shown. No changes in HR were observed across different interventions (middle). RRobs decreased significanlty ($P<0.001$) and progressively with sedation and with the implementation of NIV, consistent with spirometry findings and ventilator data. Differences between mask and nasal prongs in non-sedated (T1 vs T2) and sedated (T3 vs T4) foals were not significant. Differences during NIV were signfiicantly less that values obtained from foals without respritaory support (all $P<0.001$), but differences between different ventilator settigns were not signiicant, except as shown (T2 vs T8). Results are shown as mean (+), median (horizontal line), inter-quartile range (box) and range (whiskers), with all data points displayed.

Supplementary videos:

- Video S1 - foals displaying somnolent behaviour

  https://youtu.be/scEHaDXStPM
  https://youtu.be/kWh69_ap3iY)

- Video S2 - foals demonstrating good tolerance of NIV

  https://youtu.be/g01Ckp_m0ck
  https://youtu.be/3Svb0SNHRDk
  https://youtu.be/_jbfZwt8nOM

- Video S3 - foals demonstrating poor tolerance of NIV / nasal prongs

  https://youtu.be/0BszkY8zfUM
  https://youtu.be/CKHw18wV4Jo
  https://youtu.be/xehBl2tDQU8

Clinical case: https://youtu.be/q8oGL3o5yP4
https://youtu.be/WsKeiFL2Kes

Supplementary Table S1: Initial iteration of the behavioural ethogram for assessment of nasal prong delivery of non-invasive ventilation (NIV) in foals, as submitted in project proposal. The frequency of each identified behaviour is recorded during each observation window.

Behavioural parameters	Observation windows				
	0 – 1 min	1 – 2 min	2 – 3 min	3 – 4 min	4 – 5 min
Behaviours indicating distress					
Vocalising					
Lip smacking					
Evasive behaviours or agitation					
Seeking mare					
Defaecation					
Urination					
Other					
Behaviours indicating discomfort (with nasal prongs)					
Attempts to remove prongs against external surface					
Attempts to remove prongs with limbs / body					
Head shaking					
Respiratory distress (head extended, increased respiratory excursions)	Discontinue	Discontinue	Discontinue	Discontinue	Discontinue
Equipment requires adjustment, replacement or repositioning*					
Heart rate (bpm, from ECG)					
Resp rate (bpm, observation)					
Other comment/s or observations					

discontinue if > 5 interventions in 1 minute observation window or > 10 interventions overall, or if foal behaviour seems likely to result in self-harm (foal) or damage to equipment
